# Innate Immune Reconstitution in Humanized Bone Marrow-Liver-Thymus (HuBLT) Mice Governs Adaptive Cellular Immune Function and Responses to HIV-1 Infection

**DOI:** 10.3389/fimmu.2021.667393

**Published:** 2021-05-26

**Authors:** Wilfredo F. Garcia-Beltran, Daniel T. Claiborne, Colby R. Maldini, Meredith Phelps, Vladimir Vrbanac, Marshall E. Karpel, Katharine L. Krupp, Karen A. Power, Christian L. Boutwell, Alejandro B. Balazs, Andrew M. Tager, Marcus Altfeld, Todd M. Allen

**Affiliations:** ^1^ Department of Pathology, Massachusetts General Hospital, Boston, MA, United States; ^2^ Ragon Institute of MGH, MIT and Harvard, Massachusetts General Hospital, Cambridge, MA, United States; ^3^ Human Immune System Mouse Program, Ragon Institute of MGH, MIT, and Harvard, Cambridge, MA, United States; ^4^ Division of Medical Sciences, Harvard University, Boston, MA, United States; ^5^ Center for Immunology and Inflammatory Diseases, Massachusetts General Hospital, Boston, MA, United States; ^6^ Leibniz Institute for Experimental Virology, Hamburg, Germany

**Keywords:** innate immunity, T cells, HIV-1, HuBLT, BLT, humanized mice

## Abstract

Humanized bone marrow-liver-thymus (HuBLT) mice are a revolutionary small-animal model that has facilitated the study of human immune function and human-restricted pathogens, including human immunodeficiency virus type 1 (HIV-1). These mice recapitulate many aspects of acute and chronic HIV-1 infection, but exhibit weak and variable T-cell responses when challenged with HIV-1, hindering our ability to confidently detect HIV-1–specific responses or vaccine effects. To identify the cause of this, we comprehensively analyzed T-cell development, diversity, and function in HuBLT mice. We found that virtually all HuBLT were well-reconstituted with T cells and had intact TCRβ sequence diversity, thymic development, and differentiation to memory and effector cells. However, there was poor CD4+ and CD8+ T-cell responsiveness to physiologic stimuli and decreased TH1 polarization that correlated with deficient reconstitution of innate immune cells, in particular monocytes. HIV-1 infection of HuBLT mice showed that mice with higher monocyte reconstitution exhibited greater CD8+ T cells responses and HIV-1 viral evolution within predicted HLA-restricted epitopes. Thus, T-cell responses to immune challenges are blunted in HuBLT mice due to a deficiency of innate immune cells, and future efforts to improve the model for HIV-1 immune response and vaccine studies need to be aimed at restoring innate immune reconstitution.

## Introduction

Human immunodeficiency virus type 1 (HIV-1) first arose in Africa as a cross-species transmission event of simian immunodeficiency virus (SIV) in the 1930s (1). Today, it affects approximately 38 million adults and children worldwide, and in 2019 alone led to 1.7 million new infections and 690,000 deaths related to acquired immunodeficiency syndrome (AIDS) (Unaids Data, Vol. 2020). Currently, there are no approved vaccines to prevent or limit HIV transmission, nor are there sufficiently potent strategies to achieve a functional cure in those already infected with the virus. However, amidst our efforts to combat HIV/AIDS, a great deal has been elucidated regarding the complex interplay between the virus and host and the mechanisms employed by the virus to circumvent all aspects of the immune response (2). Consequently, host immune responses to HIV-1 have been studied with great interest in hopes of identifying key protective factors that can be harnessed for a vaccine or cure. A great deal has been learned from studies in humans, but these are hindered by our limited ability to capture individuals in acute infection, account for host genetic and environmental variability, and carry out invasive investigations of disease pathogenesis. Non-human primates are a species of close phylogenetic relationship with humans that provide an opportunity to study infections with analogous viruses that closely mimic HIV-1 infection in humans, namely, SIV and recombinant SHIV [reviewed in (3, 4)]. This has allowed for in-depth investigations into pathophysiology of disease and interventional studies. However, non-human primate studies are expensive, are complicated by host genetic diversity, and examine the pathogenesis of SIV/SHIV strains, which are ultimately different viruses from HIV-1. This has propelled the development of small-animal models that are less expensive, exhibit significantly less genetic variability, and durably support the robust replication of primary HIV isolates (5–7). Of these, the most widely studied for HIV-1 is humanized mice, also known as “human immune system” (HIS) mice.

Several humanized mouse models have been developed, mostly stemming from the introduction of severely immunodeficient mouse strains capable of achieving high levels of reconstitution with human cells. One widely used strain is NSG mice, which are non-obese diabetic (NOD) mice bearing *Prkdc^scid^* and common gamma chain null (*Il2rg^null^*) mutations (8). These lack murine lymphocytes and thus are incapable of T cell, antibody, and NK cell-mediated xenorejection. The NOD mouse strain also has an intrinsic mutation in signal-regulatory protein α (SIRPα) that binds with exceptionally high-affinity to human CD47 (9), further preventing human cell rejection by phagocytic cells. These characteristics bestow upon NSG mice an extraordinary ability to engraft human cells of various origins. These include (*i*) human peripheral blood leukocytes (HuPBL), (*ii*) human cord blood or adult hematopoietic stem cells (HuHSC), and (*iii*) human fetal liver-derived hematopoietic stem cells. When these fetal liver-derived cells are administered to NSG mice harboring fetal thymic and liver tissues implanted under the mouse renal capsule, they are also known as bone marrow-liver-thymus (BLT or HuBLT) mice (8, 10). HuPBL mice exhibit rapid human reconstitution with mature human immune cells, but rapidly develop lethal graft-versus-host disease (GvHD). HuHSC mice have long-lasting engraftment, but human T cells develop in the murine thymus and are thus restricted to murine rather than human major histocompatibility complex (MHC) molecules. These mouse-restricted human T cells have been implicated in the development of GvHD and wasting syndrome (11–13), and are, in principle, incapable of TCR : MHC-mediated cross-talk with human antigen-presenting cells such as monocytes, macrophages, dendritic cells, and B cells.

This barrier of MHC restriction was overcome with the development of HuBLT mice (14–16), which can support HLA-restricted human T-cell development *in vivo* in an autologous human thymic graft. This allows for T cells to become restricted to human MHC (also known as human leukocyte antigen, HLA) and interact with autologous human antigen-presenting cells *via* their T-cell receptor. In the context of HIV-1 infection, this in principle allows them to (*i*) be primed by autologous human monocytes and dendritic cells presenting HIV-1 peptides in the context of MHC, (*ii*) recognize MHC-presented HIV-1 peptides on HIV-1 infected CD4^+^ T cells, and (*iii*) engage with and prime cognate B cells to class switch, undergo affinity maturation, and produce anti-HIV-1 antibodies. This and other existing humanized mouse models have been used for several HIV-1-focused research studies, including antiretroviral prophylaxis (17) and therapy (18), immune cell engineering (19), broadly neutralizing antibody therapy (20–22), immunotherapies (23), reservoir purging and cure strategies (7, 18, 24), viral evolution (25, 26), transmission (27), pathogenesis (28–30), and vaccine testing (27, 31). However, studies assessing immune responses to HIV-1 infection and vaccines have been limited by variability, which calls for further characterization and improvements of HuBLT mice.

In this study, we comprehensively analyzed T-cell development, diversity, and function in HuBLT mice to identify barriers that explained deficiencies in immune responses to HIV-1 infection from that of adult humans. We found that while T-cell development and diversity is intact in HuBLT mice, there is a defect in T-cell function and responses to HIV-1 infection that correlates strongly with limited innate immune reconstitution. Thus, this study highlights that innate immune reconstitution is likely a major barrier to normal T-cell responses to HIV-1 infection in HuBLT mice.

## Methods

### Generation and Use of HuBLT Mice and Human Samples

NOD-*scid*-*Il2rg^null^* (NSG) mice (The Jackson Laboratory) were housed in a pathogen-free facility at Massachusetts General Hospital and reconstituted with human tissue as described (5). Briefly, sub-lethally irradiated mice were transplanted under the kidney capsule with 1-mm^3^ fragments of human fetal liver and thymus, made available through Advanced Bioscience Resources (ABR in Almeda, CA), and injected intravenously with purified CD34^+^ stem cells extracted from human fetal liver *via* magnetic positive selection with a CD34 MicroBead kit (Miltenyi) to generate humanized bone marrow-liver-thymus (HuBLT) mice. Human immune cells reconstitution was monitored 13 – 17 weeks post-BLT surgery and considered sufficient if >40% of lymphocytes were human CD45^+^ and >30% of human cells were CD3^+^ and reached a minimum concentration of 200 CD4^+^ T cells/μL in peripheral blood. Clinical signs of graft-versus-host disease (GvHD), such as conjunctivitis, blepharitis, alopecia, dermatitis, and weight loss were monitored for each mouse weekly. For HIV-1 infection experiments, HuBLT mice were injected *via* the intraperitoneal route with 20,000 TCID_50_ HIV_JR-CSF_. All animal experiments have been reviewed and approved by the Institutional Animal Care and Use Committee, and the use of human samples was used in accordance to protocols approved by Partners Human Research Committee and Institutional Review Board of Massachusetts General Hospital.

### Leukocyte Stimulation for Functional Assessment

Isolated leukocytes were stimulated by incubating for 4 – 6 h with phorbol 12-myristate 13-acetate (PMA) (12.5 ng/mL) and ionocymin (0.335 μM) (Cell Stimulation Cocktail used at 0.25X; eBioscience) or anti-CD3/28 Dynabeads (Life Technologies) at a bead-to-cell ratio of approximately 2:1 in the presence of brefeldin A (BioLegend) and monensin (BD Biosciences) at the manufacturers recommended concentrations. Cells were then subsequently stained with fluorescently labeled antibodies and assessed *via* flow cytometry.

### Flow Cytometric Analysis of Leukocytes From Peripheral Blood and Tissue

Direct staining of peripheral blood leukocytes was performed by addition of fluorescently labeled antibodies to whole blood and performing RBC lysis and fixation with BD FACS Lysing Solution (BD Biosciences). For stimulation experiments, peripheral blood leukocytes were isolated from whole blood by density gradient centrifugation with Histopaque (Sigma-Aldrich) to generate a layer of live mononuclear cells that was collected and washed with cell culture media consisting of 10% fetal bovine serum (Sigma-Aldrich), L-glutamine (Corning), and Primocin antibiotic (Invivogen) in RPMI-1640 (Thermo Fisher). Cells from tissues (e.g. spleen, thymus) were mechanically extracted by placing the tissue sample in a 70-μm cell strainer (Corning) in a well of a 6-well plate containing ~5 mL of cell culture media, and mashing carefully but firmly against the strainer mesh until the tissue was dissociated. The single cell suspension then underwent density gradient centrifugation to isolate live mononuclear cells.

Surface staining was performed by incubating with the corresponding antibodies at 4°C for 15 min. Cells were then washed with 2% FBS and 2 mM EDTA in PBS and fixed with 4% paraformaldehyde in PBS (Affymetrix). In experiments where CD107a surface expression was measured, the corresponding antibody was pre-incubated with the cells during stimulation for optimal staining. For measurement of cytokine-producing cells after stimulation, intracellular staining for cytokines was performed by using the BD Cytofix/Cytoperm fixation/permeabilization kit (BD Biosciences) following the manufacturer’s protocol and staining with the corresponding surface and intracellular antibodies as instructed. When applicable, anti-CD3 antibody was included in the intracellular stain to increase CD3 staining given that stimulation results in partial CD3 downregulation. Intranuclear staining for TdT was also performed by using the BD Cytofix/Cytoperm fixation/permeabilization kit. See **Supplementary Table 1** for antibodies used for flow cytometry.

Flow cytometry data was acquired on BD LSR Fortessa and analyzed using FlowJo software (version 10), and statistical analyses were performed using Microsoft Office Excel, JMP Pro 14, and GraphPad Prism 8.

### TCRβ Sequencing

RNA was extracted from isolated leukocytes using the RNeasy Plus Mini Kit (Qiagen) and QIAshredder Kit (Qiagen) following manufacturer’s instructions. 5’ rapid amplification of cDNA ends (5’ RACE) was then performed using the SMARTer RACE cDNA Amplification Kit (Clontech). cDNA was then amplified with a first round of 5’ RACE PCR using the Advantage-HF 2 Polymerase Mix (Clontech) with a 5’ universal primer mix (provided by the kit) and a gene-specific primer that recognizes all constant regions of TCRβ:

TCRβout (5’→3’): TGTGGCCAGGCACACCAGTGTGGCC

A follow-up nested PCR was performed using a nested universal primer containing an adaptor for 454 pyrosequencing (NUP) and a nested gene-specific primer that recognized all TCRβ constant regions and contained an adaptor for 454 pyrosequencing and a barcode (TCRβin):

NUP (5’→3’): CCTATCCCCTGTGTGCCTTGGCAGTCTCAGCAAGCAGTGGTATCAACGCAGAG

TCRβin (5’→3’): CCATCTCATCCCTGCGTGTCTCCGACTCAG(N)_10_GCTCAAACACAGCGACCTCGGGTGGGA

where (N)_10_ is barcoded region.

Gel band extraction for a band of approximately 450 – 500 bp was then performed using Purelink Quick Gel Extraction Kit (Invitrogen). PCR purification with QIAquick PCR Purification Kit was performed, and DNA was quantified using QUANTI-IT PicoGreen dsDNA Reagent Assay (Invitrogen) and fluorometer (Promega). Pooled PCR products were prepared for sequencing on the 454 Genome Sequencer FLX Titanium (Roche) using standard protocols (specifically, Lib L kit) and following manufacturer’s instructions. Sequence reads were analyzed using the IMGT/HighV-QUEST tool (32) and uploaded to the publicly available VDJServer (UUID: 3958818965646011925-242ac116-0001-012; https://vdjserver.org/community/3958818965646011925-242ac116-0001-012).

### Histology

Mouse tissues were freshly extracted and placed into 4% paraformaldehyde in PBS (Affymetrix) for 48 h at 4°C, and then stored in 70% ethanol at 4°C until being sent to MGH Histopathology Research Core for embedding in paraffin, sectioning, and immunofluorescent and immunohistochemical staining. Stained tissue was visualized on a TissueFAXS (TissueGnostics).

### PCR Amplification of HIV Genomes and Illumina Next-Generation Sequencing

Amplification and next-generation deep sequencing of viral genomes derived from HuBLT mice has been previously described for this data set in detail (31, 33). Additionally, this data set has been deposited in the NCBI BioProject database under accession number PRJNA552879. Briefly, HIV-1 genomes were amplified using a three-amplicon approach (Gag, Pol, and the 3’ half of the HIV-1 genome) from plasma obtained from HIV-1_JRCSF_-infected HuBLT mice 12 weeks post-infection. Libraries were then prepared using the Nextera XT DNA library preparation kit (Illumina) and were pooled and sequenced on the Illumina MiSeq platform.

### Data Availability

TCR sequencing analyses are available at VDJServer (UUID: 3958818965646011925-242ac116-0001-012; https://vdjserver.org/community/3958818965646011925-242ac116-0001-012). All 454 and Illumina next-generation sequencing data of HIV-1 genomes have been deposited in the NCBI BioProject database under accession number PRJNA552879.

## Results

### T-Cell Development Is Intact in HuBLT Mice

#### Thymopoiesis

**Figure 1:**
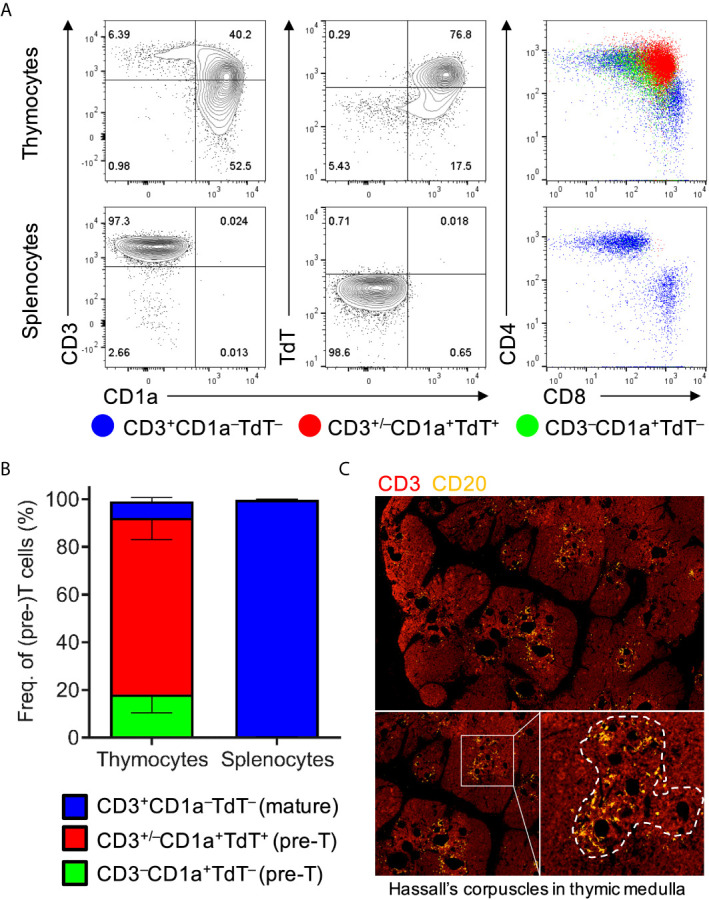
Human thymic organoids in HuBLT mice sustain thymopoiesis. **(A, B)** Thymocytes and splenocytes were extracted mechanically from fresh human thymic organoids and mouse spleens from HuBLT mice. Representative flow cytometry plots (in **A**) and aggregate data (in **B**; *n* = 6 mice from 2 distinct human tissue donors) show the frequencies of precursor T cells (CD3^–^CD1a^+^TdT^–^; green), precursor T cells undergoing TCR recombination (CD3^+/–^CD1a^+^TdT^+^; red), and mature T cells (CD3^+^CD1a^–^TdT^–^; blue). For **(B)**, stacked bars with error bars depict mean and standard deviation. **(C)** A representative immunofluorescence histology image of a thymic organoid stained with anti-CD3 (red) and anti-CD20 (orange) at three different magnifications is demonstrated; Hassall’s corpuscles in the thymic medulla containing thymic B cells is outlined with a white dotted line.

In order to characterize thymic development, thymocytes were extracted from thymic organoids and stained with human markers of T-cell development for flow cytometric analysis (34). These included CD1a, a marker of pre-T cells that is lost upon maturation, and terminal deoxynucleotide transferase (TdT), a nuclear enzyme that participates in and is virtually only expressed during V(D)J recombination. Flow cytometric analyses of thymocytes showed the characteristic abundance of double-positive (i.e. CD4^+^CD8^+^) T cells expressing both CD1a and TdT but usually lacking surface CD3, as well as the presence of mature CD4^+^ and CD8^+^ T cells that lack CD1a and TdT but express CD3 (**Figure 1A**). Analyses of thymic organoids and spleens from several mice engrafted with tissues from different donors demonstrated consistency in the development of T cells within thymic organoids, and the absence of developing T cells in the spleen (**Figure 1B**). Immunohistology of thymic organoids showed characteristic organization of thymic cortical regions as well as medullary regions containing thymic B cells surrounding Hassel’s corpuscles at the cortico-medullary junction (**Figure 1C**). Thus, thymopoiesis and human thymic architecture is intact in HuBLT mice.

#### Repertoire Diversity

**Figure 2:**
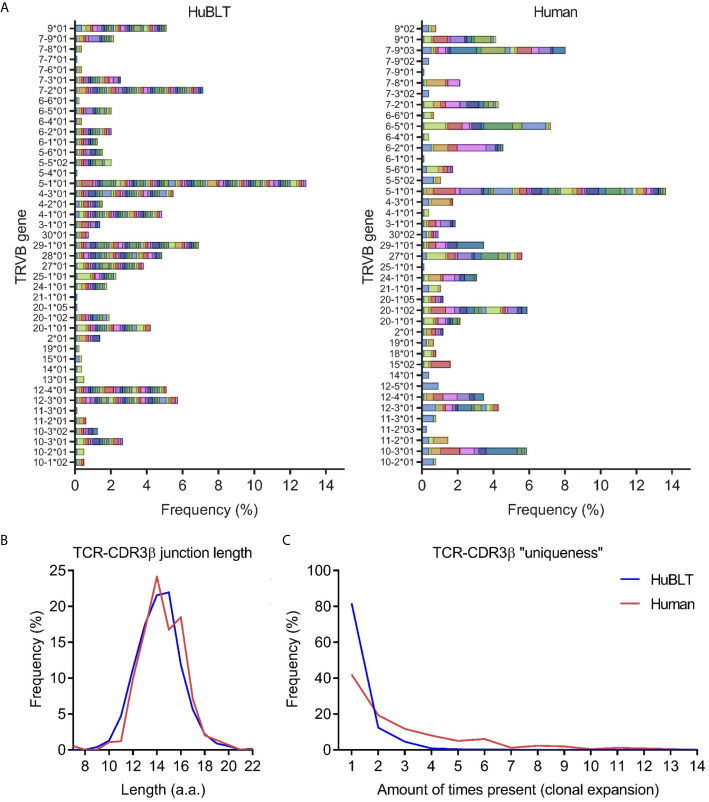
TCR diversity in HuBLT mice is comparable to adult humans. **(A)** TCRβ deep sequencing was performed on leukocytes from a HuBLT mouse spleen and an adult human peripheral blood donor. Deep sequencing reads were analyzed for TRBV gene usage and CDR3β sequence, amino-acid length, and “uniqueness”. The frequency of sequences for HuBLT mouse (left panel) and adult human (right panel) samples grouped by TRBV usage are presented as a histogram (normalized to the total number of sequence reads obtained). Each CDR3β clone is individually color-coded to visualize clonal expansion. **(B, C)** A histogram of CDR3β junction length (in **B**) and a histogram of CDR3β “uniqueness” (in **C**) of HuBLT mouse (blue) and adult human (red) samples are presented. For each sample, ~800 TCRβ transcript reads of ~500 bp in length were obtained.

Given existing literature suggesting that fetal T-cell repertoires are limited in diversity (35), we aimed to determine the diversity of T cells in HuBLT mice *via* TCR sequencing. Prior studies have demonstrated that the complementarity-determining region 3 of the TCRβ chain (CDR3β) best captures the full diversity of a polyclonal T-cell population as compared to sequencing of TCRα (36, 37). Thus, we developed a deep sequencing protocol that amplified and sequenced TCRβ transcripts from bulk T cells in an unbiased fashion. To prevent biased amplification of TCRβ transcript bearing specific Vβ regions, we used 5’ rapid amplification of cDNA ends (5’ RACE) technology to forgo use of pooled Vβ region primers [the most commonly used strategy in other studies (38)]. Deep sequencing with read lengths of approximately 500 bp was performed to capture full TCRβ transcripts without the need for assembly. Results demonstrated that TCRβ diversity was comparable, if not greater, than that found in adult human peripheral blood (**Figure 2A**), which is in line with previous studies showing diverse Vβ usage in HuBLT mice (39). CDR3β lengths in HuBLT mice showed a Gaussian-like normal distribution (**Figure 2B**), which is thought to arise from randomly generated indels in the CDR3 region during V(D)J recombination (40), and had more unique CDR3β sequences (**Figure 2C**), which characteristic of “naïve” immune systems. On the other hand, adult human T cells showed a bimodal distribution of CDR3β length (**Figure 2B**) and had more oligoclonal expansion of specific T cells secondary to immune challenges (**Figure 2C**) **(** 40). From this, we concluded that T-cell repertoires in HuBLT mice are diverse and likely able to recognize a wide array of antigens.

#### Phenotypic Subsets

**Figure 3:**
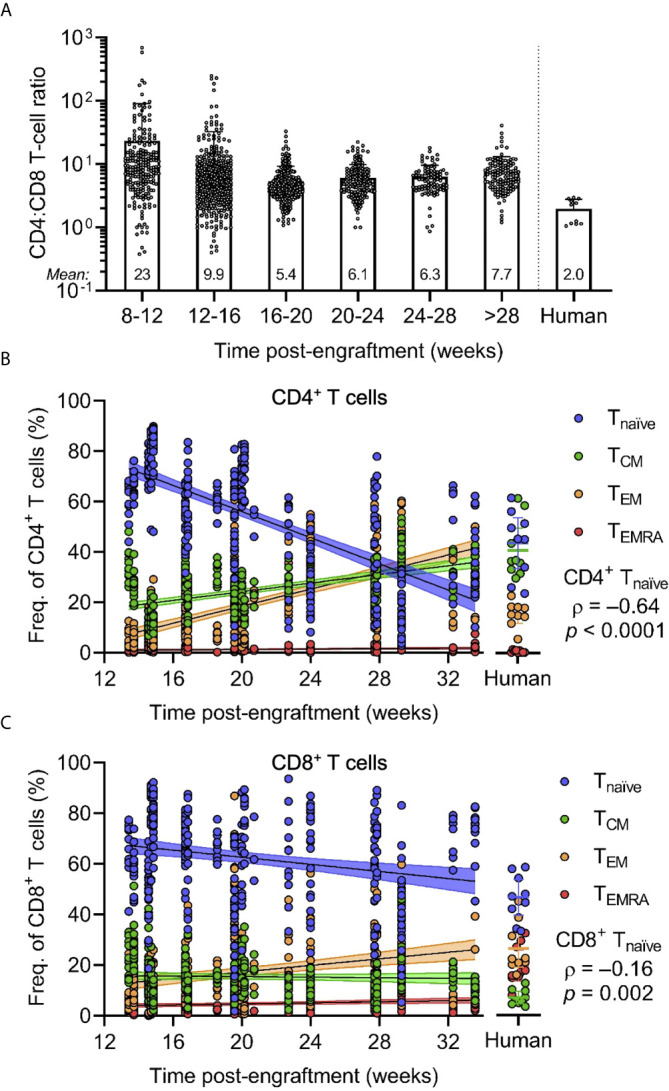
CD4/CD8 T-cell ratios and naïve T cell frequencies are increased in HuBLT mice but change with age. **(A)** Peripheral blood leukocytes from HuBLT mice (*n* = 685 mice from 33 distinct human tissue donors) and healthy adult human donors (*n* = 11) were analyzed *via* flow cytometry to assess for CD4:CD8 T-cell ratios at different times post-engraftment. Data was binned at 4-week intervals, with mean CD4:CD8 T-cell ratios for each bin depicted at the bottom of each bin bar; some mice had repeat measurements at multiple time points. **(B, C)** Peripheral blood leukocytes from HuBLT mice (*n* = 329 mice from 17 distinct human tissue donors) and healthy adult human donors (*n* = 9) were analyzed *via* flow cytometry to assess for frequencies of naïve (T_naïve_; CD45RA^+^CCR7^+^; blue), central memory (T_CM_; CD45RA^–^CCR7^+^; green), effector memory (T_EM_; CD45RA ^–^CCR7^–^; orange), and effector memory re-expressing CD45RA (T_EMRA_; CD45RA^+^CCR7^–^; red) cells within CD4^+^ T cells (in **B**) and CD8^+^ T cells (in **C**) at different times post-engraftment. For **(B, C)**, linear regression (black line) with 95% confidence intervals (colored shade) for each population is depicted for visualization, as well as Spearman coefficients (ρ) with corresponding *p* values for the indicated T_naïve_ population.

To further investigate the extent of T-cell development and differentiation, we assessed the frequency of CD8^+^ versus CD4^+^ and naïve versus memory T cells. Flow cytometric analyses of the peripheral blood of HuBLT mice generated from different tissue donors at different times post-engraftment demonstrated that CD4^+^ T cells are often the most abundant human cells in peripheral blood of HuBLT mice, with very high CD4:CD8 T-cell ratios (**Figure 3A**). HuBLT mice at 8-12, 12-16, 16-20, 20-24, 24-28, and >28 weeks post-engraftment exhibited the following CD4:CD8 T-cell ratios (mean ± s.d.): 23 ± 67, 9.9 ± 22.7, 5.4 ± 3.9, 6.1 ± 3.7, 6.3 ± 3.3, and 7.7 ± 5.4, respectively. Adult human peripheral blood, however, had a CD4:CD8 T-cell ratio of 2.0 ± 0.8. Interestingly, high CD4:CD8 T-cell ratios in HuBLT mice have been previously described (7) and reflect the peripheral blood of early gestational age fetuses, which exhibits lymphocytic predominance and high CD4:CD8 T-cell ratios of 4-5 approximately (35).

Phenotypic analysis of naïve and memory T-cell subsets was performed by staining for CD45RA and CCR7. Peripheral blood T cells of HuBLT mice contained naïve (T_naïve_; CD45RA^+^CCR7^+^), central memory (T_CM_; CD45RA^–^CCR7^+^), effector memory (T_EM_; CD45RA^–^CCR7^–^), and effector memory re-expressing CD45RA (T_EMRA_; CD45RA^+^CCR7^–^) T-cell subsets that varied within the limits of healthy adult human peripheral blood (**Figures 3B, C**). However, there was a significant correlation between the age of HuBLT mice and the frequency of memory T cells, with older HuBLT mice bearing an overrepresentation of memory T cells as compared to young BLT mice that have a predominance of T_naïve_ cells (age versus CD4^+^ T_naïve_: ρ = –0.64, *p* < 0.0001; age versus CD8^+^ T_naïve_: ρ = –0.16, *p* = 0.002). Of note, newborns also exhibit a predominance of T_naïve_ cells that decreases with age (41). Consequently, we determined that human T cells in HuBLT mice indeed possess the ability to mature and differentiate into memory and effector subsets.

### T-Cell Function Is Dependent on Innate Immune Reconstitution

#### Stimulus Responsiveness

**Figure 4:**
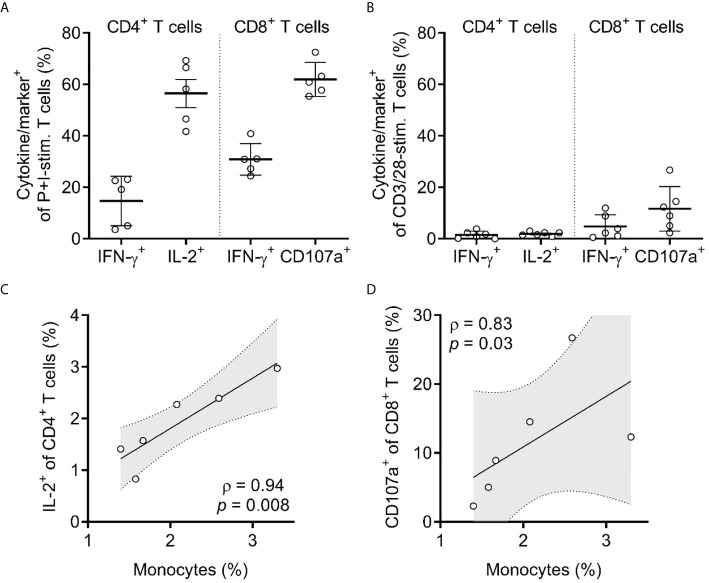
T cells in HuBLT mice have impaired responsiveness that correlates with inadequate innate immune reconstitution. **(A, B)** CD4^+^ and CD8^+^ T cells from peripheral blood leukocytes of HuBLT mice (*n* = 6 mice from 2 distinct human tissue donors) were assessed *via* flow cytometry for expression of the indicated cytokines or markers (i.e. IFN-γ, IL-2, or CD107a) after stimulation with either PMA and ionomycin (P+I-stim., in **A**) or anti-CD3/28 Dynabeads (CD3/28-stim., in **B**). Although not shown, values for unstimulated cells were negligible (<0.5%). **(C, D)** Correlations were made between monocyte frequencies (percent of CD14^+^ cells of all hCD45^+^ cells) and the frequencies of responding (IL-2^+^) CD4^+^ T cells (in **C**) and responding (CD107a^+^) CD8^+^ T cells (in **D**). For **(C, D)**, linear regression (black line) with 95% confidence intervals (gray shade) is depicted for visualization, as well as Spearman coefficients (ρ) with corresponding *p* values.

Having confirmed that the development and differentiation of T cells was intact, we sought to assess their function in response to a variety of stimuli and immune challenges. We stimulated bulk peripheral blood leukocytes with phorbol 12-myristate 13-acetate and ionomycin (P+I), which mimic diacylglycerol and calcium release downstream of TCR and co-receptor signaling, and performed surface and intracellular staining. This demonstrated that P+I potently induced IL-2 production in CD4^+^ T cells, degranulation of CD8^+^ T cells, and IFN-γ production from both (**Figure 4A**). However, when assessing the responsiveness of peripheral blood T cells to anti-CD3/28 Dynabeads (CD3/28), a more physiological stimulus that crosslinks TCR and co-stimulatory receptor CD28, we found an exceptionally blunted response by T cells, with only minimal capacity to produce IL-2 in CD4^+^ T cells or degranulate in CD8^+^ T cells (**Figure 4B**), which is in line with previous studies that report a defect with anti-CD3 stimulation (42, 43). When attempting to correlate the response magnitude of CD3/28-stimulated T cells to several biological parameters, we found that there was a substantial positive correlation between the frequency of IL-2^+^ CD4^+^ T cells and the frequency of monocytes (CD14^+^ cells) in peripheral blood (ρ = 0.94, *p* = 0.008) (**Figure 4C**), which also held true for degranulating (CD107a^+^) CD8^+^ T cells (ρ = 0.83, *p* = 0.03) (**Figure 4D**). These findings demonstrate that although HuBLT human T cells are intrinsically capable of performing effector T-cell functions (as seen by P+I stimulation), that are defects in response to stimulation *via* the TCR and CD28 that correlate to monocyte reconstitution. This is congruent with prior studies in HuBLT mice using HIV-1-specific CAR-T cells, which showed that CD14^+^ monocyte reconstitution correlated with CAR-T-cell expansion (44). Altogether, these data gave insight into a link between innate immune reconstitution and T-cell responses in HuBLT mice.

#### T-Cell Polarization

**Figure 5:**
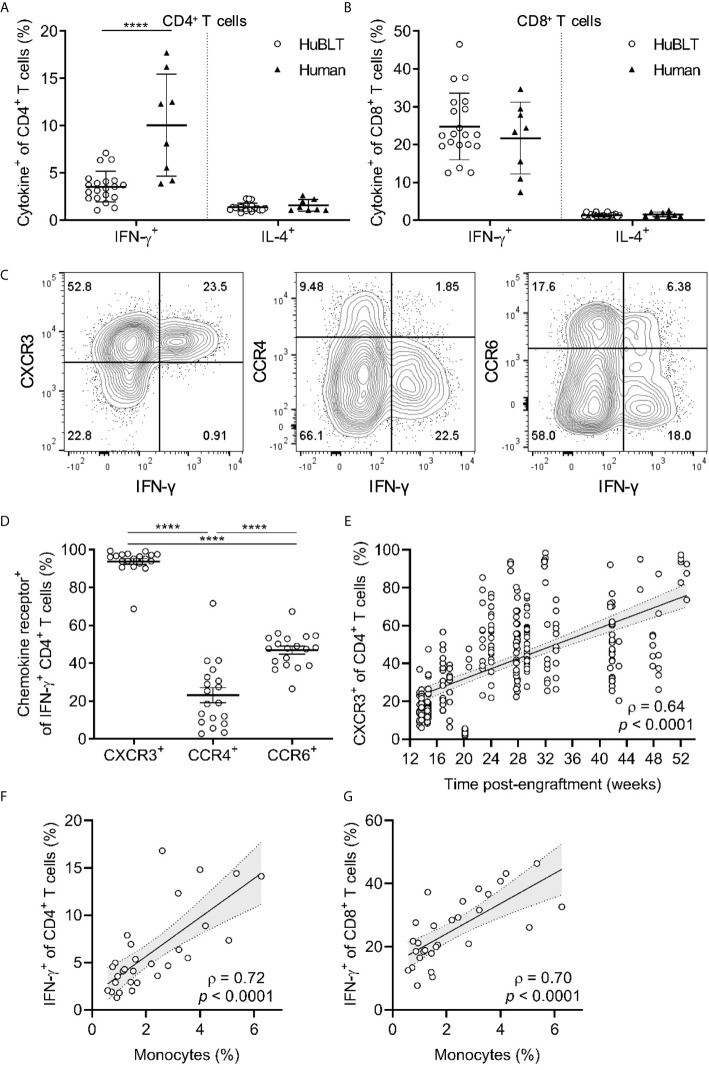
T_H_1 polarization in HuBLT mice is impaired but improves with innate immune reconstitution and age. **(A, B)** Peripheral blood leukocytes from HuBLT mice (*n* = 20 from 1 human donor tissue; empty circles) and healthy adult human donors (*n* = 8, black triangles) were stimulated with PMA and ionomycin and assessed *via* flow cytometry for expression of IFN-γ and IL-4 in CD4^+^ T cells (in **A**) and CD8^+^ T cells (in **B**). **(C, D)** PMA- and ionomycin-stimulated CD4^+^ T cells from the peripheral blood of HuBLT mice were assed for IFN-γ production and expression of the indicated chemokine receptors (CXCR3, CCR4, and CCR6). Representative flow plots (in **C**) and aggregate data (in **D**, *n* = 19 mice from 6 distinct human tissue donors) are presented. **(E)** Peripheral blood CD4^+^ T cells from HuBLT mice (*n* = 282 mice from 18 distinct human tissue donors) were analyzed for CXCR3 expression as a surrogate marker for T_H_1 polarization at different times post-engraftment. **(F, G)** Monocyte frequencies (percent of CD14^+^ cells of all hCD45^+^ cells) were correlated to PMA- and ionomycin-stimulated IFN-γ expression in CD4^+^ T cells (in **F**) and CD8^+^ T cells (in **G**) in HuBLT mice (*n* = 29 mice from 2 distinct human tissue donors >12 weeks post-engraftment). For **(A**, **B**, **D)**, lines with error bars depict mean and standard deviation, and **** denotes *p* < 0.0001 after performing a Wilcoxon signed rank test. For **(E–G)**, linear regression (black line) with 95% confidence intervals (gray shade) are depicted for visualization, as well as Spearman coefficients (ρ) with corresponding *p* values.

Interestingly, CD4^+^ T cells that produced IFN-γ in response to P+I stimulation were found to be lower than what would be expected for adult human peripheral leukocytes (**Figure 4A**) **(** 45). We hypothesized that this may be due to impaired T_H_1 polarization of CD4^+^ T cells in HuBLT mice. Consequently, we assessed the polarization states of CD4^+^ T cells in HuBLT mice by measuring the frequency of T_H_1 and T_H_2 cells using functional assays that measure cytokine production in response to P+I stimulation. We found that there were significantly decreased frequencies of T_H_1 (IFN-γ^+^) cells in the peripheral blood of HuBLT as compared to that of adult humans (IFN-γ^+^ CD4^+^ T-cell frequencies of 3.5% ± 1.6% in HuBLT mice and 10.0% ± 5.4% in adult humans, *p <*0.0001) (**Figure 5A**). T_H_2 (IL-4^+^) cell frequencies were not significantly increased in HuBLT mice. Although IFN-γ^+^ frequencies were higher in CD8^+^ T cells than CD4^+^ T cells, they did not differ between HuBLT mice and human donors (**Figure 5B**).

Studies have reported that chemokine receptor expression can be used as a surrogate for T_H_ polarization status, namely, that CXCR3 expression correlates with IFN-γ production (T_H_1 polarization) (46), CCR4 expression correlates with IL-4 production (T_H_2 polarization) (46), and CCR6 expression correlates with IL-17 production (T_H_17 polarization) (47). We explored whether these associations held true for T_H_1-polarized cells in HuBLT mice to use these surrogate markers in longitudinal studies of polarization status across different batches of mice. Indeed, we found that CXCR3 positivity enriched for IFN-γ-producing CD4^+^ T cells (significantly more than for IL-4- or IL-17-producing CD4^+^ T cells) (**Supplementary Figure 1**), and that almost all IFN-γ^+^ CD4^+^ T cells expressed CXCR3 (significantly more than CCR4 or CCR6) (**Figures 5C, D**). This indicated that CXCR3 was an adequate surrogate marker for T_H_1 cells in HuBLT mice as has been shown in adult humans. Upon assessment of CXCR3^+^ T cells across several cohorts of HuBLT mice of varying ages, we found that T_H_1 polarization increased with age (**Figure 5E**), indicating that human T cells in HuBLT indeed retain the capacity to differentiate into T_H_1 polarized cells, but that there was delay characterized by a defect that was pronounced early after reconstitution. We hypothesized that similar to previous findings, this defect may be due to variable and inadequate innate immune reconstitution. Upon further analyses, we found that monocyte reconstitution positively correlated with T_H_1 frequencies (ρ = 0.72, *p* < 0.0001) (**Figure 5F**) and IFN-γ^+^ CD8^+^ T-cell frequencies (ρ = 0.70, *p* < 0.0001) (**Figure 5G**), indicating that innate immune reconstitution was critical for T-cell differentiation into a T_H_1-polarized effector-cell fate in HuBLT mice. Of note, it is known that IL-12 produced by professional antigen presenting cells such as monocytes, macrophages, and dendritic cells is necessary for T_H_1 differentiation and IFN-γ production, providing a mechanistic link between innate immune reconstitution and T_H_ polarization status in HuBLT mice.

### T-Cell Responses and Viral Evolution in HIV-1-Infected HuBLT Mice Correlate With Innate Immune Reconstitution

**Figure 6:**
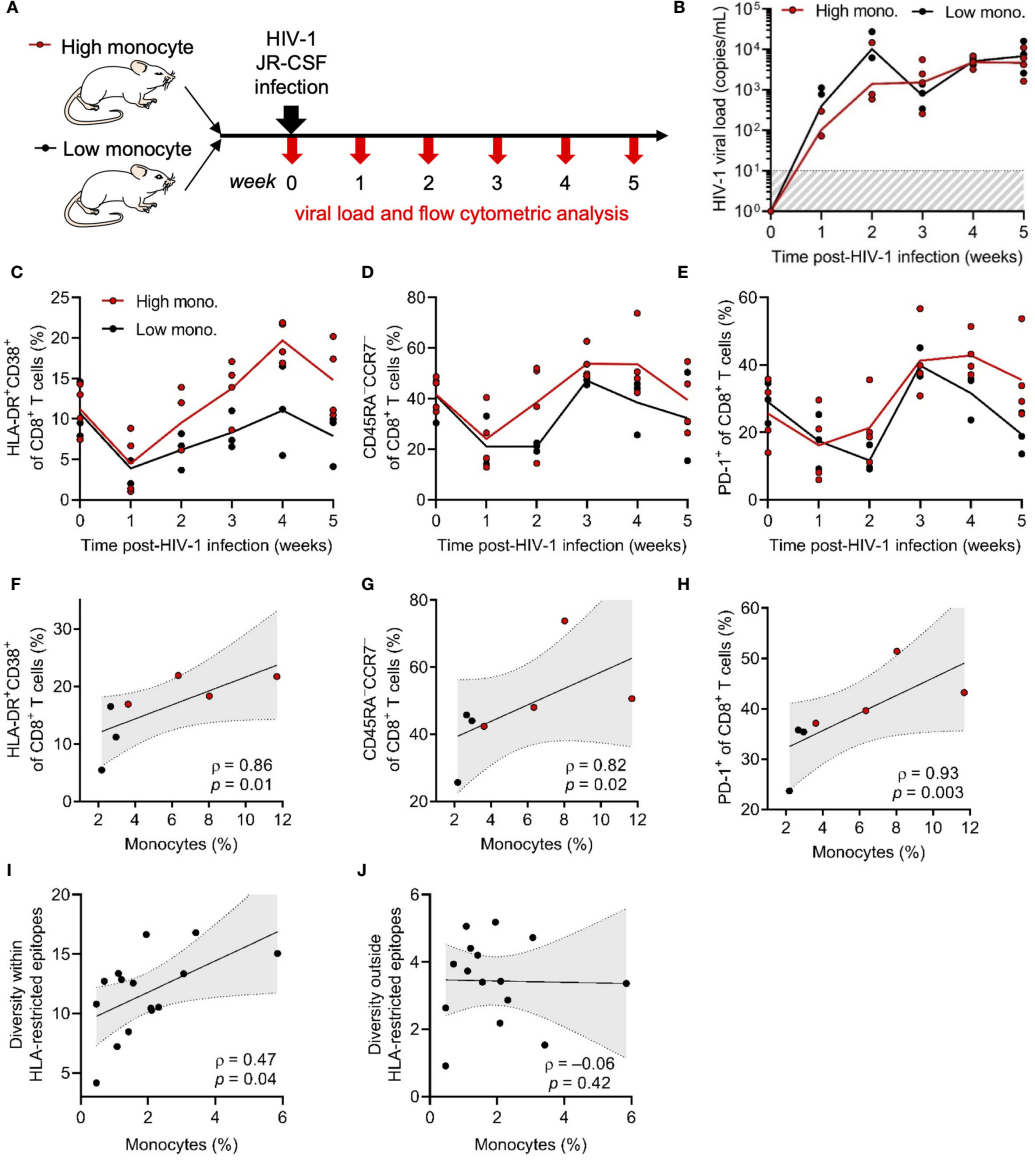
CD8^+^ T cell responses to HIV-1 infection correlate with innate immune reconstitution. **(A)** Represented is a schematic of HuBLT mice (*n* = 7 mice from 1 human donor tissue) grouped into a high peripheral blood monocyte frequency group (high mono.; red circles) and a low monocyte frequency group (low mono.; black circles). Mice were infected with HIV-1_JR-CSF_ (2 x 10^5^ TCID_50_, intraperitoneal route) and CD8^+^ T-cell phenotypes were longitudinally assessed *via* flow cytometry. **(B)** HIV-1 viremia was assessed longitudinally by qRT-PCR specific for *gag* p24 in each HuBLT mouse; lines represent group means over time. **(C–E)** CD8^+^ T cell activation (HLA-DR^+^CD38^+^; in **C**), differentiation into effector memory cells (CD45RA^–^CCR7^–^; in **D**), and expression of the activation/exhaustion marker PD-1 (in **E**) was assessed at different times post infection; lines represent group means over time. **(F–H)** Monocyte reconstitution (percent of CD14^+^ cells of all hCD45^+^ cells) was correlated to peak CD8^+^ T-cell responses (week 4). **(I, J)** In a separate experiment, HuBLT mice (*n* = 15 mice from 1 human donor tissue) were infected with HIV-1_JR-CSF_ (2 x 10^5^ TCID_50_, intraperitoneal route). Whole viral genome sequencing was performed at 12 weeks post-infection to assess evolving codons, which were defined as positions in which at least one mouse showed >10% diversity compared to HIV-1_JR-CSF_ consensus. HLA-restricted epitopes were defined as those predicted to be restricted by the HLA class I type (or HLA class I supertype as defined by Sidney et al., 2008 *BMC Immunol*) of the human donor tissue. The diversity within and outside HLA-restricted epitopes for each mouse was calculated by averaging the percent variation for all evolving codons contained within or outside HLA-restricted epitopes, respectively (as has been previously described in Claiborne et al., 2019 *J Virol*). Monocyte frequencies (percent CD14^+^ cells of all hCD45^+^ cells) for each mouse were correlated to diversity within (in **H**) and outside (in **I**) HLA-restricted epitopes. For **(F–J)**, linear regression (black line) with 95% confidence intervals (gray shade) are depicted for visualization, as well as Spearman coefficients (ρ) with corresponding *p* values.

To assess whether T-cell responsiveness was affected by innate immune reconstitution *in vivo*, we grouped HuBLT mice as bearing “high” and “low” monocyte frequencies (based on the median of the cohort) and challenged with HIV-1 (**Figure 6A**). While all mice became viremic (**Figure 6B**), CD8^+^ T cell activation (HLA-DR^+^CD38^+^), differentiation into effector memory (CD45RA^–^CCR7^–^), and expression of exhaustion markers (PD-1^+^) was higher in the “high” monocyte frequency group (**Figures 6C–E**), with monocyte frequencies correlating to the peak of these CD8^+^ T-cell responses (ρ = 0.86, *p* = 0.01; ρ = 0.82, *p* = 0.02; and ρ = 0.93, *p* = 0.003, respectively) (**Figures 6F–H**). This data suggests that the frequency of innate immune cells is rate-limiting for the *in-vivo* priming of CD8^+^ T-cell responses in HuBLT mice.

In order to draw a link between innate immune reconstitution and pathogen-targeted immune responses, we evaluated the effect of monocyte reconstitution on HIV-1 sequence diversity within predicted HLA-restricted epitopes as a metric of CD8^+^ T cell-mediated immune pressure (31, 48). We also evaluated the influence of monocyte frequencies on HIV-1 sequence diversity outside of predicted HLA-restricted epitopes, which in principle are not amenable to CD8^+^ T cell-mediated immune pressure. Remarkably, we found that monocyte frequencies significantly correlated with diversity in HLA-restricted epitopes (ρ = 0.47, *p* = 0.04) (**Figure 6I**), while there was no significant association with diversity outside these regions (**Figure 6J**). This supports the notion that innate immune reconstitution drives CD8^+^ T-cell responses to HIV-1 infection and in turn exerts immune pressure and drives HIV-1 sequence evolution, although further studies directly assessing T-cell responses to HIV-1 epitopes are needed.

## Discussion

### Innate Immunity Is Critical to Eliciting Adaptive Immunity

The requirement of innate antigen-presenting cells for maintaining and priming T-cell immunity is a well-known immunological concept that has been re-emphasized by our comprehensive analysis of T-cell immunology in HuBLT mice. We find that the model achieves the desired endogenous thymic development, TCR repertoire diversity, and generation of T-cell subsets comparable to that of humans, suggesting that efforts at improving these processes are likely not required in the model. However, poor reconstitution of monocytes significantly correlated to defects in T-cell function across multiple contexts, including functional maintenance, T_H_ polarization, priming, and anti-viral responses during HIV-1 infection. The latter finding was a striking observation that monocyte reconstitution not only significantly correlated with CD8^+^ T cell activation, but also with CD8^+^ T cell-mediated immune pressure as measured by viral evolution within (but not outside) HLA-restricted epitopes, suggesting that human-like HIV-1-specific T-cell responses can arise in HuBLT mice when all necessary elements are present. Prior experiments in immunocompetent mice have demonstrated that *in-vivo* depletion or absence of mouse dendritic cells hinders priming of CD8^+^ T cells (49). In addition, it has been shown that mouse inflammatory monocytes orchestrate CD8^+^ T-cell activation during microbial infections (50). Our study makes evident that these same requirements exist at baseline as well as during anti-viral immune responses in HuBLT mice.

### Human-Mouse Incompatibilities

Defective reconstitution of innate immune cells has been previously described in humanized mouse models (10, 27, 51–56). Hematopoietic stem cell transplant (HSCT) patients and HuBLT mice exhibit similar reconstitution dynamics post-transplant, with characteristic myeloid, B-cell, and T-cell “waves” in most settings (57). However, in contrast to HSCT patients, our data and previous literature demonstrate that the myeloid wave in HuBLT mice does not persist. The early wave of myeloid cells results from short-term repopulating CD34^+^ precursor cells committed to the myeloid lineage differentiating into mature cells (57). However, there is a defect in long-term myeloid reconstitution that is likely due to inadequate support of myeloid differentiation in hematopoietic stem cells in HuBLT. This notion of defective myelopoiesis is supported by the known lack of cross-reactivity between murine and human cytokines and growth factors necessary for human myeloid cell development (**Table 1**) (58).

**Table 1:** Cross-reactivity of human and mouse cytokines and their receptors.

Cytokine	Cross-reactivity (ligand → receptor)		Relevant Function
	mouse → human	human → mouse	
**SCF**	↓2.5-fold	none	Myelopoiesis
**IL-3**	none	none	Myelopoiesis
**GM-CSF**	none	none	Myelopoiesis
**M-CSF**	none	full	Myelopoiesis
**G-CSF**	active	full	Myelopoiesis
**EPO**	↓2.5-fold	active	Myelopoiesis
**TPO**	full	active	Myelopoiesis
**FLT-3L**	full	full	Myelo-/Lymphopoiesis
**IL-6**	none	active	Myelo-/Lymphopoiesis
**IL-1α/β**	active	active	Lymphopoiesis
**IL-2**	none	active	Lymphopoiesis
**IL-4**	none	none	Lymphopoiesis
**IL-7**	active	active	Lymphopoiesis
**IL-12**	active	none	Lymphopoiesis
**TNFα**	active	active (not TNFR2)	Lymphopoiesis
**TGF-β1**	full	full	Lymphopoiesis
**CXCL12**	active	active	Lymphopoiesis, Bone marrow homing
**CCL19/21**	full	(n.d.)	T-cell zone homing
**CXCL13**	↓100-fold	(n.d.)	B-cell zone homing
**TSLP**	none	none	Lymph node genesis

‘full’ = approximately 100% cross-reactivity has been reported; ‘active’ = cross-reactivity has been reported but not quantitated; ‘none’ = negligible activity has been reported; ‘(n.d.)’ = no data readily available.

### Efforts to Enhance Innate Immune Reconstitution in Humanized Mice

Non-cross-reactivity of these critical factors has led many investigators to develop strategies that introduce human cytokines and growth factors by various methods including (*i*) recombinant protein injections, (*ii*) hydrodynamic transfection (59), (*iii*) generation of genetically modified [e.g. transgenic (60–62) and knock-in (63–66)] mouse strains, including the well-known NSG-SGM3 and MISTRG mice, and (*iv*) introduction of cytokine-encoding gene therapy vectors (67, 68). These have the goal of offsetting the cross-species barriers and enhancing human hematopoiesis and immune function. In this study, we identified deficient innate immune reconstitution as a significant model-intrinsic defect, and identified this as the major barrier to predictable adaptive immune responses in HuBLT mice. These findings pave the road towards rational improvement of the model by directing efforts to restoring innate immune reconstitution and better recapitulate immune responses to HIV-1 and ultimately test candidate vaccines.

## Data Availability Statement

The datasets presented in this study can be found in online repositories. The names of the repository/repositories and accession number(s) can be found below: NCBI BioProject, accession no: PRJNA552879.

## Ethics Statement

The animal study was reviewed and approved by Massachusetts General Hospital Institutional Animal Care and Use Committee.

## Author Contributions

WG-B, DC, CM, MK, MA, and TA designed experiments. WG-B, DC, CM, MP, VV, MK, KK, and KP conducted experiments. KP, CB, AT, MA, and TA provided significant and insightful contributions to design of experiments. VV, AT, MA, and TA provided significant resources. WG-B and DC wrote the paper with contributions from all other authors. All authors contributed to the article and approved the submitted version.

## Funding

This work was supported by the US National Institute of Health (P01-AI104715 to MA, and TA; F31AI116366 to WG-B; 1F32AI136750 to DC; 5T32AI007529-21A1 to MK), the National Institute of General Medical Sciences (T32GM007753 to WG-B), and the Ragon Institute of MGH, MIT and Harvard. AB is supported by the National Institutes for Drug Abuse (NIDA) Avenir New Innovator Award DP2DA040254, the MGH Transformative Scholars Program as well as funding from the Charles H. Hood Foundation. This independent research was supported by the Gilead Sciences Research Scholars Program in HIV. The funder was not involved in the study design, collection, analysis, interpretation of data, the writing of this article or the decision to submit it for publication.

## Disclaimer

The content is solely the responsibility of the authors and does not necessarily represent the official views or policies of the National Institute of General Medical Sciences, the National Institutes of Health, or the Department of Health and Human Services, nor does mention of trade names, commercial products, or organizations imply endorsement by the US Government.

## Conflict of Interest

The authors declare that the research was conducted in the absence of any commercial or financial relationships that could be construed as a potential conflict of interest.
