# Supplementary material for: Innate Immune Reconstitution in Humanized Bone Marrow-Liver-Thymus (HuBLT) Mice Governs Adaptive Cellular Immune Function and Responses to HIV-1 Infection
Source: Front Immunol. 2021 May 26;12:667393. doi: 10.3389/fimmu.2021.667393 (PMC8189152; doi:10.3389/fimmu.2021.667393)
Supplement: Supplementary file 1 [file DataSheet_1.pdf]

Supplemental Figure 1

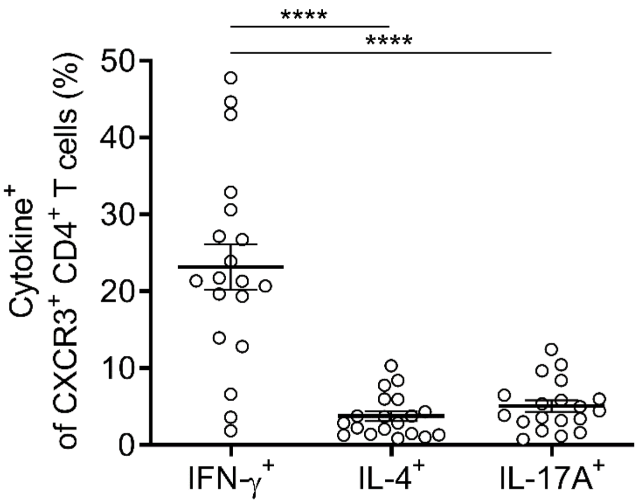

Supplemental Figure 1: CXCR3 serves as a surrogate marker for T_H1 polarization in HuBLT mice. PMA- and ionomycin-stimulated $CD4^+$ T cells from the peripheral blood of HuBLT mice (n = 19 mice from 6 distinct human donors tissues, same as from **Figure 5D**) were assessed for expression of the chemokine receptor CXCR3, which is used as a surrogate marker for T_H1 expression in humans, and production of the indicated cytokines as more definitive markers of polarization status. The frequency of IFN-γ^+, IL-4^+, and IL-$17A^+$ cells within $CXCR3^+$ $CD4^+$ T cells is plotted for each mouse. One-way ANOVA with Tukey's multiple comparisons test was performed; **** denotes $p < 0.0001$.

Supplemental Table 1

Supplementary Table 1: Antibodies used for HuBLT flow cytometry				
**Marker**	**Fluorophore**	**Antibody Clone**	**Company**	**µL/50 µL**
hCD45	Alexa Fluor 700	HI30	BioLegend	1
CD3	PerCP-Cy5.5	UCHT1	BioLegend	1
CD4	BV785	RPA-T4	BioLegend	1
CD8	APC-Cy7	RPA-T8	BioLegend	1
CD45RA	PE-Cy7	HI100	BioLegend	1
CCR7	BV421	150503	BD Biosci.	2
CD38	BV605	HIT2	BioLegend	2
HLA-DR	BV510	L243	BioLegend	1
PD-1	APC	EH12.2H7	BioLegend	2
CD14	BUV395	MφP9	BD Biosci.	2
IL-2	BV421	5344.111	BD Biosci.	1
CD107a	PE-Cy7	H4A3	BioLegend	2
IFN-γ	AF647	4S.B3	BioLegend	1
IL-4	PE	8D4-8	BioLegend	1
IL-17A	BV605	BL168	BioLegend	1
CXCR3	FITC	G025H7	BioLegend	1
CCR4	BV421	L291H4	BioLegend	1
CCR6	PE-Cy7	11A9	BD Biosci.	1
CD1a	PE-Cy5	HI149	BioLegend	2.5
TdT	FITC	E17-1519	BD Biosci.	5
